# Higher Alu Methylation Levels in Catch-Up Growth in Twenty-Year-Old Offsprings

**DOI:** 10.1371/journal.pone.0120032

**Published:** 2015-03-25

**Authors:** Kittipan Rerkasem, Prakasit Rattanatanyong, Amaraporn Rerkasem, Antika Wongthanee, Kittipong Rungruengthanakit, Ampica Mangklabruks, Apiwat Mutirangura

**Affiliations:** 1 Department of Surgery, Faculty of Medicine, Chiang Mai University, Chiang Mai, Thailand; 2 The Research Institute for Health Sciences, Chiang Mai University, Chiang Mai, Thailand; 3 Center of Excellence of Molecular Genetics of Cancer and Human Diseases, Department of Anatomy, Faculty of Medicine, Chulalongkorn University, Bangkok, Thailand; 4 Department of Internal Medicine, Faculty of Medicine, Chiang Mai University, Chiang Mai, Thailand; Centre Hospitalier Universitaire Vaudois, FRANCE

## Abstract

Alu elements and long interspersed element-1 (LINE-1 or L1) are two major human intersperse repetitive sequences. Lower Alu methylation, but not LINE-1, has been observed in blood cells of people in old age, and in menopausal women having lower bone mass and osteoporosis. Nevertheless, Alu methylation levels also vary among young individuals. Here, we explored phenotypes at birth that are associated with Alu methylation levels in young people. In 2010, 249 twenty-years-old volunteers whose mothers had participated in a study association between birth weight (BW) and nutrition during pregnancy in 1990, were invited to take part in our present study. In this study, the LINE-1 and Alu methylation levels and patterns were measured in peripheral mononuclear cells and correlated with various nutritional parameters during intrauterine and postnatal period of offspring. This included the amount of maternal intake during pregnancy, the mother’s weight gain during pregnancy, birth weight, birth length, and the rate of weight gain in the first year of life. Catch-up growth (CUG) was defined when weight during the first year was >0.67 of the standard score, according to WHO data. No association with LINE-1 methylation was identified. The mean level of Alu methylation in the CUG group was significantly higher than those non-CUG (39.61% and 33.66 % respectively, P < 0.0001). The positive correlation between the history of CUG in the first year and higher Alu methylation indicates the role of Alu methylation, not only in aging cells, but also in the human growth process. Moreover, here is the first study that demonstrated the association between a phenotype during the newborn period and intersperse repetitive sequences methylation during young adulthood.

## Introduction

DNA methylation is an epigenetic mark directly on CpG dinucleotide sequences [1]. The majority of DNA methylation in the human genome is on intersperse repetitive sequences (IRS). IRS methylation plays a crucial role in cellular phenotypes, controlling genomic integrity as well as gene expression [2]. Reduction of genomic methylation can lead to genomic instability [1], relating to endogenous DNA double strand break repair [3]. Genomic instability is one of the hallmark characteristics of cancer and aging cells. Global hypomethylation also alters gene expression. For example, long interspersed element-1 (LINE-1 or L1) can regulate the degree of gene expression by adjusting intragenic LINE-1 methylation level [4,5]. Here in this study we evaluated whether IRS methylation during young adulthood would be associated with phenotypes during the new born period.

Alu elements are human abundant IRS, presenting up to 300,000 copies in the human genome [1]. Lower Alu methylation has been observed in blood cells of people during old age [6], and in menopausal women having lower bone mass and osteoporosis [7], familial breast cancer [8], gastric cancer [9], and chronic lymphocytic leukemia [10]. On the other hand, higher Alu methylation has been reported in various conditions such as colorectal cancer [11], insulin resistance [12], cardiovascular risk, including hypertension/diabetes [13], and systemic lupus erythematosus [14]. No change was found in many conditions, such as exposure to pollutants, including metals and particulate air pollution [15], breast cancer [16], and prenatal arsenic exposure [17].

Most of LINE-1s are truncated. Only approximately 2 thousand copies contain 5’UTR, where methylated CpG were studied [1]. Many studies of blood cells reported lower methylation of LINE-1 in pollution exposure [9,18–20], smoking in patients with Parkinson’s disease [21], increased oxidative stress [22], and several cancers [23], whereas higher methylation of LINE-1 was detected in early colorectal cancer [11], malignant melanoma [24]. Recently some studies reported the association between intrauterine and early life insult and epigenetic with the levels of line-1 methylation [17]. For example intrauterine exposure to higher levels of arsenic was positively associated with DNA methylation in LINE-1 in umbilical cord blood [17]. Also lower LINE-1 methylation is related to development of adiposity in 553 boys, aged 5–12 years [25].

Here we investigated the correlation between phenotypes of the perinatal period with IRS methylation during young adulthood. In 2010, we invited volunteers who had participated as newborns for birth weight and nutrition during a pregnancy study in 1990 (Chiang Mai Low Birth Weight Study-CMLBWS) [26]. We found that offsprings with history of intrauterine growth retardation (IUGR) such as poor intrauterine nutrition, mother with pregnancy induced hypertension (PIH) were associated significantly with rapid weight gain (catch-up growths) in the first year of life than those offsprings without IUGR [27]. Also these IUGR offsprings with abundant postnatal nutrition were significantly associated with catch-up growths (CUG) during the first year of life. These CUG had been reported previously, but was associated with metabolic and non-communicable diseases, such as high body fat deposition [28], increased blood pressure [29], and diabetes [30]. However, the precise mechanism of this association is still unknown. The epigenetic memory was proposed to be a molecular mechanism [31]. During in utero, or early postnatal development, short term changes through environmental affect could permanently change gene expression, and consequently organ development at a time of extreme vulnerability.

We chose to evaluate DNA methylation, not only its levels, but also its patterns. Changes in DNA methylation of IRS is not homogenous. Previously, we demonstrated not only a general, but also locus specific influence of LINE-1 methylation [32]. Therefore, in some situations methylation of different IRS loci were not harmoniously changed. For example, both hypo and hypermethylated LINE-1 loci can be discovered in smoke-exposed oral epithelial [33]. Currently, there are two commonly used techniques: pyrosequencing [1], and Combine Bisulfite Restriction Analysis (COBRA) [34].Both techniques precisely measure methylation levels. Unfortunately, pyrosequencing can only measure DNA methylation levels. Therefore, herein this study, we chose to evaluate LINE-1 and Alu methylation levels and patterns by COBRA.

## Materials and Methods

In 2010, we invited adolescents whose mothers had participated in a birth weight and nutrition during pregnancy study in 1990 [26]. In brief, the 1990 study recruited 2184 pregnant women with gestational age < 24 weeks. Researchers followed up every subject’s antenatal care up to delivery. The study recorded demographic data, anthropometric data, socioeconomic data. During delivery, the birth weight, birth length, and placental weight were also recorded. As a follow-up, every three months in the first year of the child’s life, their weight was also recorded. Maternal diet intake was assessed by two methods. First, the 24-hour food recall method was used during the initial interview. Each mother was asked to recall all food consumed during the previous day and to estimate quantities in ordinary measures or servings. Then all details were calculated by using Thai Food Tables [35]. The amount of food was calculated as energy, protein, fat and carbohydrate at each of the three trimesters: weeks 10–12 (first trimester), weeks 22–24 (second trimester), and weeks 32–34 (third trimester).The food frequency questionnaire (FFQ) was used to assess the frequency of consumption of 34 foods in the previous month. Nutrient intakes in the FFQ were validated against the 24-hour food recall method.

In 2010, all 2184 offspring were invited to participate in the study. Their histories, physical exams, and blood samples were collected to determine their current general condition. The test consisted of the participants sitting quietly in a room at the clinic for at least 20 minutes. Twice, at intervals of 5–10 minutes, their blood pressure was measured on the left arm at heart level. Anthropometric measurements were performed. While wearing indoor clothes, the participant’s height, weight, and waist circumference measurement were taken. Each participant filled out questionnaires on various cardiovascular risk factors, smoking experience, and past medical history. The participants fasted at least 12 hours before attending the study, and then venous blood samples were collected. Total cholesterol and plasma glucose were measured using the Beckman Coulter analyzer (UnicelDxc 800, Fullerton, California, USA).

### COBRA LINE-1 and COBRA Alu

Also blood samples were collected and extracted to measure the level of LINE-1 and Alu methylation [36]. DNA extraction was performed by standard phenol chloroform extraction protocol. All DNA samples were treated with sodium bisulfite essentially following guidelines provided (EZ DNA Methylation-Gold Kit, Zymo research corp, Orange, CA, USA). For COBRA LINE-1, the bisulfate-treated DNA was subjected to 40 PCR cycles with LINE-1-F (5’-CGTAAGGGGTTAGGGAGTTTTT-3’) and LINE-1-R (5’-RTAAAACCCTCCRAACCAAATATAAA-3’) primers at an annealing temperature of 50°C. For COBRA Alu, the bisulfite-treated DNA was subjected to 40 cycles of PCR with two primers, Alu-F (5’-GGCGCGGTGGTTTACGTTTGTAA-3’) and Alu-R (5’TTAATAAAAACGAAAT TTCACCATATTA ACCAAAC-3’) at an annealing temperature of 53°C. After PCR amplification, the LINE-1 amplicons (160 bp) were digested with *TaqI* and *TasI* in NEB buffer 3 (New England Biolabs, Ontario, Canada), while the Aluamplicons (117 bp) were digested with *TaqI* in *TaqI* buffer (MBI Fermentas, Burlington, Canada). Both digestion reactions were incubated at 65°C overnight. The LINE-1 and Alu element digested products were then electrophoresed on an 8% non-denaturing polyacrylamide gel and stained with the SYBR green nucleic acid gel stain (Gelstar, Lonza, Rockland, ME, USA). Distilled water was used as negative control. All experiments were performed in duplicate. DNA samples from HeLa, Jurkat and Daudi cell lines were used as positive controls in every experiment and to standardize interassay variation [1].

Both COBRA LINE-1 and COBRA Alu detected methylation status of two CpG dinucleotides [36]. Therefore, COBRA can report four IRS methylation patterns, hypermethylation (^m^C^m^C) when both of the CpGs of the same locus were methylated. Hypomethylation (^u^C^u^C) when both of the CpGs of the same locus were unmethylated. We also reported two partial methylation pattern (^m^C^u^C and ^u^C^m^C). Both methylation level and pattern were reported in percentage number. For methylation levels we reported the percentage of methylated CpG. For methylation pattern, percentage numbers of loci of each pattern were determine. Detail analysis of LINE-1 and Alu methylation levels and patterns were the same as recently reported [36].

### Statistical analysis

We analyzed the association between the level of LINE-1 and Alu methylation with various nutritional parameters, both intrauterine factors and early postnatal period. Since our study in LINE-1 and Alu methylation was conducted in blood sampling 20 years later, we therefore analyzed current parameters and epigenetic levels to see the difference between perinatal risk factors and current risk factors. The small for gestational age (SGA) defined as weight <10 percentile of gestational age [37], and the history of CUG in weight during the first year of life, defined the weight >0.67 standard score according to WHO data [38]. For the dichotomous data, an independent sample *t*-test was performed to determine differences between LINE-1 and Alu element methylation patterns. The continuous data were analyzed for the correlation with Pearson method. All P value was corrected for multiple comparisons (fault discovery rate—Simmes method). The data was presented in mean and standard deviation. Analysis was performed by STATA for Windows version 13.0. The significant levels quoted were two-sided and P < 0.05 was considered statistically significant.

### Ethics statement

This study was conducted according to the guidelines laid down in the Declaration of Helsinki, and all procedures involving human subjects were approved by the Human Experimentation Committee, Research Institute for Health Sciences, Chiang Mai University, Chiang Mai, Thailand (Project Number 17/52). Written informed consent was obtained from all subjects together with their mothers and participants’ anonymity was preserved.

## Results

249 participants, who were offspring in CMLBWS, were recruited in this study. There were 103 males (41.4%), 27 current smokers (10.8%), 49 SGA, and 45 CUG. Mothers in CMLBWS, on average, had a normal range of BMI (Table 1). During delivery phase, the mean birth weight of offspring was 2814.54 grams, which was not in a low birth weight range.

**Table 1:** Baseline data of participants during pregnancy and delivery period (in 1990) and during follow up period in 2010 study.

Baseline item	Mean ± standard deviation
***Pregnancy and delivery period***	
Mothers age (yr) during pregnancy	26 ± 4.63
Body mass index at recruitment in study	21.26 ± 2.50
Birth weight (gram)	2814.54 ± 452.07
Birth length (cm)	47.89 ± 4.61
Gestational age (months)	38.90 ± 1.98
Age of mother during delivery (years)	26.20 ± 4.69
Placental weight (gm)	556 ± 111.57
Placental diameter (cm)	19.20 ± 2.64
***Follow up period***	
Age of offsprings (months)	246.06 ± 5.63
Waist circumference (cm)	77.25 ± 1.30
Body mass index	21.71 ± 4.80
Plasma cholesterol (mg/dl)	167.13 ± 31.93
Fasting blood sugar (mg/dl)	83.66 ± 13.27
Systolic blood pressure (mmHg)	115.23 ± 12.93
Diastolic blood pressure (mmHg	73.71 ± 10.80

**Fig 1:**
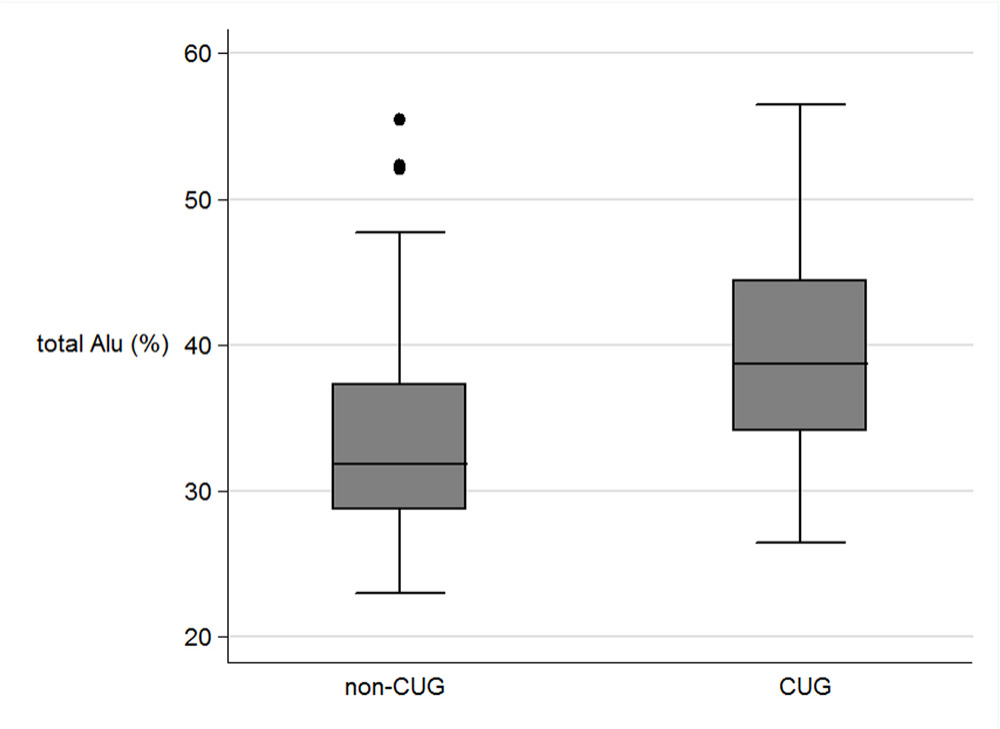
The boxplots of the total Alu methylation (%) between non catch up growth group (non-CUG) and catch up growth (CUG).

**Fig 2:**
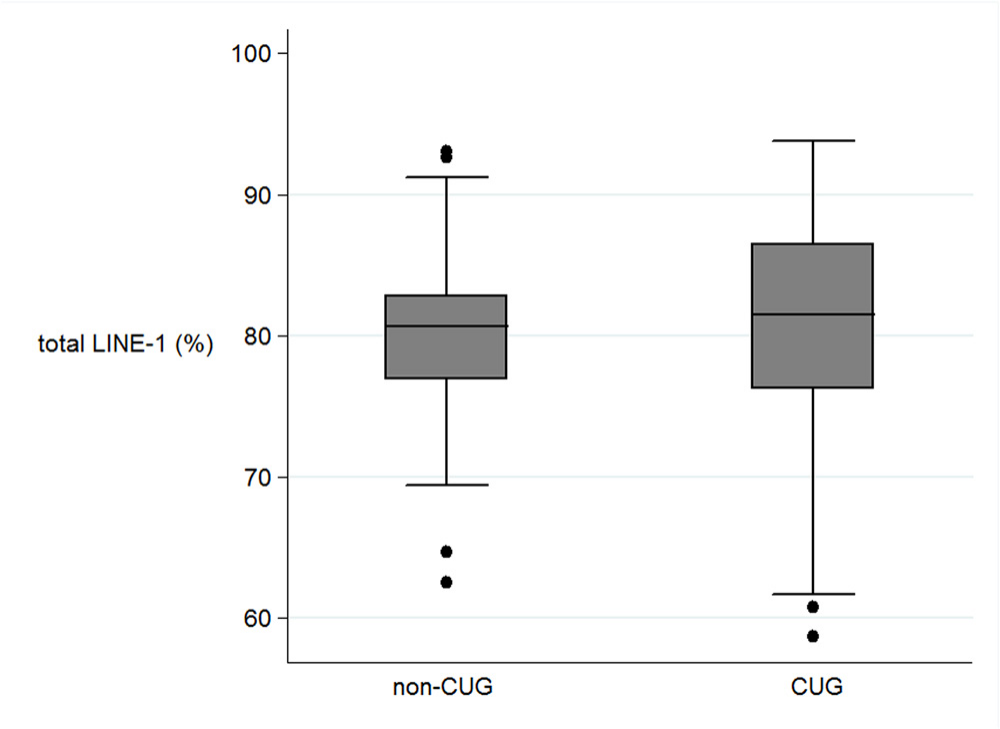
The boxplots of the LINE-1 methylation (%) between non catch up growth group (non-CUG) and catch up growth (CUG).

In the perinatal parameters, there were significant correlations only between CUG and non-CUG in the levels of methylation in the percentage of total Alu, Alu_UU, and Alu_MM (Table 2). The mean level of total Alu_methylation in the CUG group was marked higher than those not in CUG (39.61% and 33.66% respectively, P < 0.0001) (Fig. 1). In contrast, the mean level of unmethylated loci (Alu_UU) in the CUG group was considerably lower than those in non-CUG group (37.39% and 44.85%, P<.0001 respectively). In contrast, there was no significant association between the levels of Alu_methylation with other perinatal parameters. The LINE-1 methylation levels were not correlated with any perinatal parameters (Fig. 2).

**Table 2:** The comparison of percentage of Alu (above panel) methylation and the percentage of LINE-1 methylation (below panel) in participants who are absence or presence the following risk factors: catch up growth, small for gestational age, male and smoking history.

*Type of methylation*	Total Alu		Alu_UU		Alu_MM		Alu_UM		Alu_MU	
***Intrauterine factor/early postnatal factors***										
***Catch up growth history***	Absence	Presence	Absence	Presence	Absence	Presence	Absence	Presence	Absence	Presence
*Mean*	33.66	39.61	44.85	37.39	12.16	16.60	23.75	26.04	19.23	19.97
*SD*	6.99	7.22	8.76	8.71	9.07	8.58	6.95	7.11	6.06	6.55
*p-values control the FDR(simes)*	<0.00001		<0.00001		0.0233		0.1858		0.7849	
***Small for gestational age history***	Absence	Presence	Absence	Presence	Absence	Presence	Absence	Presence	Absence	Presence
*Mean*	36.32	33.91	40.50	44.61	13.14	12.44	25.56	24.06	20.80	18.90
*SD*	7.67	7.72	8.78	9.36	11.00	8.31	7.89	6.73	6.89	5.54
*p-values control the FDR(simes)*	0.1428		0.0700		0.6999		0.3723		0.1428	
***Gender of off spring***	Female	Male	Female	Male	Female	Male	Female	Male	Female	Male
*Mean*	34.63	34.06	43.20	44.62	12.47	12.74	25.04	23.38	19.30	19.26
*SD*	7.92	7.53	9.66	8.93	8.89	8.95	7.30	6.43	5.56	6.32
*p-values control the FDR(simes)*	0.8233		0.6373		0.9136		0.3820		0.9574	
***Smoking history***	Absence	Presence	Absence	Presence	Absence	Presence	Absence	Presence	Absence	Presence
*Mean*	34.07	35.27	44.07	42.99	12.21	13.53	24.30	23.49	19.42	19.99
*SD*	7.55	7.75	9.37	8.72	8.53	9.79	6.85	5.50	5.68	6.77
*p-values control the FDR(simes)*	0.6251		0.6251		0.6251		0.6251		0.6251	
***Type of methylation***	**Total LINE- 1**		**LINE- 1_MM**		**LINE- 1_UU**		**LINE- 1_MU**		**LINE- 1_UM**	
***Intrauterine factor/early postnatal factors***										
***Catch up growth history***	Absence	Presence	Absence	Presence	Absence	Presence	Absence	Presence	Absence	Presence
*Mean*	79.87	79.74	49.09	50.49	7.86	8.35	22.28	20.19	20.78	20.98
*SD*	5.52	8.95	12.20	18.26	5.40	6.21	9.35	10.10	14.19	15.46
*p-values control the FDR(simes)*	0.9410		0.7849		0.7849		0.4542		0.9410	
***Small for gestational age history***	Absence	Presence	Absence	Presence	Absence	Presence	Absence	Presence	Absence	Presence
*Mean*	79.24	80.21	48.95	49.53	9.27	7.31	23.11	21.90	18.67	21.26
*SD*	6.23	5.96	13.14	12.42	6.22	4.99	9.03	9.02	13.63	13.25
*p-values control the FDR(simes)*	0.4470		0.7718		0.0990		0.5007		0.3723	
***Gender of off spring***	Female	Male	Female	Male	Female	Male	Female	Male	Female	Male
*Mean*	80.11	79.88	49.91	48.72	8.24	6.93	21.54	22.98	20.31	21.37
*SD*	5.73	6.42	12.09	13.18	5.69	4.61	8.39	9.81	13.14	13.65
*p-values control the FDR(simes)*	0.9136		0.8233		0.3820		0.6373		0.8233	
***Smoking history***	Absence	Presence	Absence	Presence	Absence	Presence	Absence	Presence	Absence	Presence
*Mean*	79.69	80.48	48.82	49.94	7.86	6.61	21.57	23.38	21.76	20.06
*SD*	5.98	6.96	12.46	14.23	5.54	3.60	8.92	9.50	13.54	12.72
*p-values control the FDR(simes)*	0.6251		0.6251		0.6251		0.6251		0.6251	

There was no significant correlation between the levels of Alu_methylation and LINE-1 methylation with various intrauterine parameters (during pregnancy), including maternal intake during 3 trimesters (carbohydrate, protein, fat, energy, weight gain), birth weight and placental weight (Table 3). Similarly no correlation was found between Alu_methylation and LINE-1 methylation with early postnatal factors.

**Table 3:** The correlation analysis between the percentage of Alu and LINE-1 methylation in various nutritional factors during pregnancy and delivery.

Type of methylation	Total Alu	Alu_UU	Alu_MM	Alu_UM	Alu_MU	Total LINE-1	LINE-1_MM	LINE-1_UU	LINE-1_MU	LINE-1_UM
***Intrauterine factor/early postnatal factors***										
**bmi in first visit of mothers**										
Correlation, r	0.0868	-0.1361	0.0079	0.1834	-0.0131	0.0025	0.0165	0.0588	-0.0620	0.0031
p-values control the FDR(simes)	0.6217	0.1895	0.9687	0.0500	0.9687	0.9687	0.9687	0.7134	0.7134	0.9687
**Gestational age at delievery**										
Correlation, r	0.0061	0.0307	0.0430	-0.0218	-0.0883	0.0797	0.0437	-0.1459	-0.1139	0.0938
p-values control the FDR(simes)	0.9260	0.8015	0.7340	0.8233	0.4222	0.4222	0.7340	0.2150	0.3675	0.4222
**Mother age when pregnancy**										
Correlation, r	0.1340	-0.1308	0.0956	0.0357	0.0215	-0.0758	-0.0496	0.0675	0.0603	-0.0209
p-values control the FDR(simes)	0.2300	0.2300	0.4853	0.7341	0.7446	0.5737	0.6236	0.5737	0.5737	0.7446
**Mother weight gain in 1st trim**										
Correlation, r	-0.2221	0.1445	-0.2911	0.0495	0.0008	-0.0719	-0.0911	-0.0730	0.0495	0.0809
p-values control the FDR(simes)	0.9188	0.9188	0.9188	0.9188	0.9972	0.9188	0.9188	0.9188	0.9188	0.9188
**Mother weight gain in 2nd trim**										
Correlation, r	0.0359	0.0313	0.0939	-0.0664	-0.1127	0.0282	0.0194	-0.0134	-0.0977	0.0543
p-values control the FDR(simes)	0.8295	0.8295	0.5313	0.8012	0.5313	0.8295	0.8364	0.8364	0.5313	0.8030
**Mother weight gain in 3rd trim**										
Correlation, r	0.0069	-0.0298	-0.0191	0.0597	0.0054	-0.0026	0.0399	0.1415	-0.1234	-0.0075
p-values control the FDR(simes)	0.9681	0.9681	0.9681	0.9681	0.9681	0.9681	0.9681	0.2835	0.2835	0.9681
**maternal protein intake in 1st trim**										
Correlation, r	0.1792	-0.3042	-0.0243	0.3200	0.2015	-0.1120	-0.0679	0.0569	0.1524	-0.0596
p-values control the FDR(simes)	0.4560	0.1070	0.8576	0.1070	0.4427	0.6782	0.7490	0.7490	0.5154	0.7490
**maternal protein intake in 2nd trim**										
Correlation, r	-0.0781	0.0889	-0.0426	-0.0523	-0.0090	0.0771	0.1000	0.0220	-0.0971	-0.0347
p-values control the FDR(simes)	0.6140	0.6140	0.8016	0.8016	0.9062	0.6140	0.6140	0.8533	0.6140	0.8016
**maternal protein intake in 3rd trim**										
Correlation, r	0.0351	0.0047	0.0662	-0.0471	-0.0516	0.0649	0.0316	-0.0599	-0.0524	0.0277
p-values control the FDR(simes)	0.7752	0.9494	0.7752	0.7752	0.7752	0.7752	0.7752	0.7752	0.7752	0.7752
**maternal carbohydrate intake in 1st trim**										
Correlation, r	0.0455	-0.1566	-0.1002	0.2867	0.0863	-0.0482	-0.0179	0.0986	0.1776	-0.1482
p-values control the FDR(simes)	0.8188	0.6780	0.7476	0.3060	0.7476	0.8188	0.8950	0.7476	0.6780	0.6780
**maternal carbohydrate intake in 2nd trim**										
Correlation, r	0.0130	-0.0545	-0.0335	0.0541	0.0717	-0.0548	-0.0362	0.0292	-0.0124	0.0326
p-values control the FDR(simes)	0.8679	0.8679	0.8679	0.8679	0.8679	0.8679	0.8679	0.8679	0.8679	0.8679
**maternal carbohydrate intake in 3rd trim**										
Correlation, r	0.0667	-0.0562	0.0586	0.0261	-0.0319	0.0584	0.0239	-0.0744	-0.0437	0.0346
p-values control the FDR(simes)	0.7371	0.7371	0.7371	0.7371	0.7371	0.7371	0.7371	0.7371	0.7371	0.7371
**maternal fat intake in 1st trim**										
Correlation, r	-0.1108	-0.0070	-0.2159	0.1875	0.1165	-0.2166	-0.1785	0.1022	0.0731	0.0820
p-values control the FDR(simes)	0.6420	0.9589	0.4600	0.4600	0.6420	0.4600	0.4600	0.6420	0.6543	0.6543
**maternal fat intake in 2nd trim**										
Correlation, r	-0.0856	0.0317	-0.1138	0.0983	0.0020	-0.0075	-0.0097	-0.0065	0.0657	-0.0357
p-values control the FDR(simes)	0.8757	0.9797	0.8757	0.8757	0.9797	0.9797	0.9797	0.9797	0.9422	0.9797
**maternal fat intake in 3rd trim**										
Correlation, r	-0.1703	0.2038	-0.0872	-0.1354	-0.0260	0.0059	-0.0040	-0.0109	-0.0217	0.0223
p-values control the FDR(simes)	0.0975	0.0500	0.5855	0.2130	0.9557	0.9557	0.9557	0.9557	0.9557	0.9557
**Amount of energy intake in 1st trim**										
Correlation, r	0.0310	-0.1641	-0.1361	0.3124	0.1248	-0.1082	-0.0672	0.1172	0.1745	-0.1053
p-values control the FDR(simes)	0.8189	0.5444	0.5444	0.1800	0.5444	0.5444	0.6880	0.5444	0.5444	0.5444
**Amount of energy intake in 2nd trim**										
Correlation, r	-0.0230	-0.0237	-0.0635	0.0658	0.0541	-0.0340	-0.0152	0.0283	-0.0078	0.0094
p-values control the FDR(simes)	0.9169	0.9169	0.9169	0.9169	0.9169	0.9169	0.9169	0.9169	0.9169	0.9169
**Amount of energy intake in 3rd trim**										
Correlation, r	-0.0078	0.0339	0.0214	-0.0367	-0.0421	0.0534	0.0186	-0.0692	-0.0468	0.0398
p-values control the FDR(simes)	0.9152	0.8831	0.8831	0.8831	0.8831	0.8831	0.8831	0.8831	0.8831	0.8831
**brithweight**										
Correlation, r	-0.0985	0.1054	-0.0604	0.0283	-0.1105	0.1267	0.0767	-0.1694	-0.1095	0.0693
p-values control the FDR(simes)	0.2233	0.2170	0.3984	0.6673	0.2170	0.2170	0.3270	0.0750	0.2170	0.3466
**birthlegth**										
Correlation, r	-0.1064	0.0905	-0.0887	0.0533	-0.0713	0.0487	0.0102	-0.1007	-0.0204	0.0438
p-values control the FDR(simes)	0.5120	0.5120	0.5120	0.6491	0.6170	0.6491	08809	0.5120	0.8487	0.6491
**Placental weight**										
Correlation, r	-0.0935	0.0415	-0.1196	0.0860	0.0118	-0.0427	-0.0417	-0.0056	-0.0587	0.0809
p-values control the FDR(simes)	0.5340	0.6724	0.5340	0.5340	0.9320	0.6724	0.6724	0.9320	0.6724	0.5340

trim = trimester

**Fig 3:**
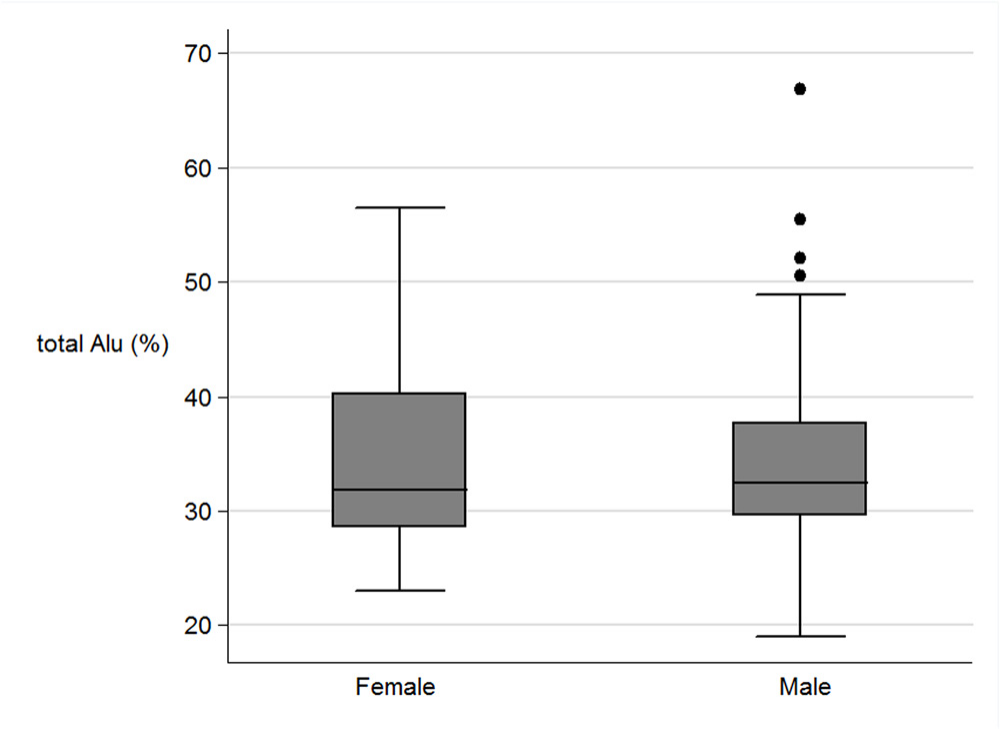
The boxplots of the total Alu methylation (%) between female and male.

**Fig 4:**
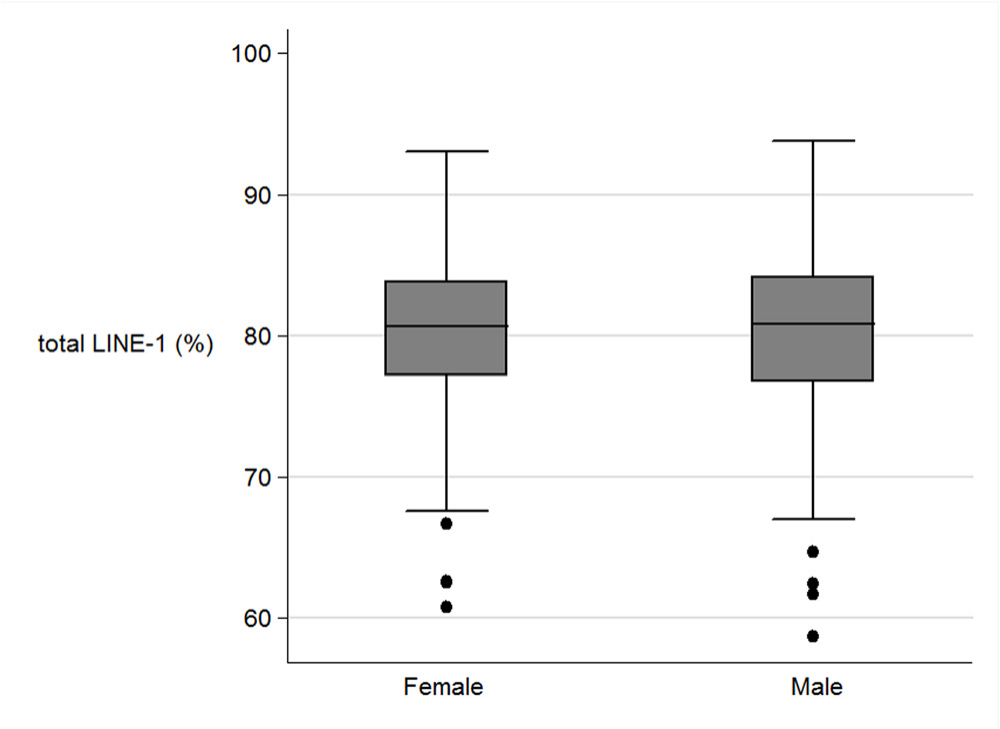
The boxplots of the LINE-1 methylation (%) between female and male.

There was no significant correlation between Alu_methylation and LINE-1 methylation with current factors such as gender, age, BMI, waist circumference, plasma cholesterol, fasting glucose and blood pressure (Table 4) (Figs. 3, 4). When authors analysed the association between CUG and non-CUG with the perinatal data, CUG group had higher incidence of pregnancy induce hypertension of mother during pregnancy than those in non-CUG (9.8% and 0.3% respectively, P = 0.04). Also maternal fat intake in the first trimester in CUG group was significant lower than those in non-CUG group (P = 0.03). When authors explored further on the association between CUG and non-CUG with current factors, waist circumference in CUG group was significantly higher than those in non-CUG group (78.6 cm and 74.9 cm respectively, P = 0.04). The mean BMI in CUG group was higher than those in non- CUG group (22.5 cm and 20.7 cm respectively, P = 0.05).

**Table 4:** The correlation analysis between the percentage of Alu and LINE-1 methylation in various current risk factors during follow up study (2010).

Type of methylation	Total Alu	Alu_uu	Alu_MM	Alu_UM	Alu_MU	Total LINE-1	LINE-1_MM	LINE-1_UU	LINE-1_MU	LINE-1_UM
***Factors in follow up study 2010***										
**Age at recent study**										
Correlation, r	0.0503	-0.0050	0.0815	-0.0294	-0.0805	0.0056	-0.0010	-0.0492	-0.0083	0.0261
p-values control the FDR(simes)	0.9881	0.9881	0.9881	0.9881	0.9881	0.9881	0.9881	0.9881	0.9881	0.9881
**Body mass index in recent study**										
Correlation, r	0.0307	-0.0788	-0.0295	0.0497	0.1114	-0.0459	-0.0716	-0.1211	-0.0350	0.1391
p-values control the FDR(simes)	0.6541	0.5224	0.6541	0.6541	0.2997	0.6541	0.5224	0.2845	0.6541	0.2845
**Waist circumference in recent study**										
Correlation, r	-0.0013	-0.0324	-0.0364	0.0155	0.0885	-0.0536	-0.0819	-0.1282	-0.0360	0.1532
p-values control the FDR(simes)	0.9839	0.7789	0.7789	0.9049	0.5010	0.7789	0.5010	0.2230	0.7789	0.1620
**Plasma cholesterol in recent study**										
Correlation, r	-0.0063	-0.0361	-0.0498	0.0482	0.0755	-0.0183	-0.0035	0.0604	-0.0228	-0.0034
p-values control the FDR(simes)	0.9589	0.9589	0.9589	0.9589	0.9589	0.9589	0.9589	0.9589	0.9589	0.9589
**Fasting glucose in recent study**										
Correlation, r	-0.0478	0.0655	-0.0145	-0.0858	0.0186	0.0545	0.0671	0.0758	-0.0958	-0.0287
p-values control the FDR(simes)	0.6713	0.6428	0.8270	0.6428	0.8270	0.6582	0.6428	0.6428	0.6428	0.8174
**Systolic blood pressure in recent study**										
Correlation, r	0.0679	-0.0244	0.0925	-0.0917	0.0077	-0.0123	-0.0162	-0.0653	-0.0523	0.0766
p-values control the FDR(simes)	0.6138	0.9072	0.6138	0.6138	0.9072	0.9072	0.9072	0.6138	0.6892	0.6138
**Diastolic blood pressure in recent study**										
Correlation, r	0.0773	-0.0369	0.0959	-0.0158	-0.0677	-0.0465	-0.0463	0.0155	-0.1551	0.1423
p-values control the FDR(simes)	0.6015	0.7194	0.4850	0.8133	0.6084	0.6697	0.6697	0.8133	0.1270	0.1270

trim = trimester

## Discussion

This study evaluated the association between Alu and LINE-1 methylation of 20-year-old individuals with various phenotypes during their babies period (intrauterine and early postnatal period). Whereas no association with LINE-1 methylation was identified, the correlation between the history of CUG in the first year, and higher Alu methylation,was demonstrated. Interestingly, Alu, but not LINE-1 hypomethylation, is associated with aging, and also disease phenotype due to aging, such as osteoporosis [6][7]. These two evidences suggest that methylation of different IRSs possess different functions. Alu methylation plays a role in cell growth and prevents cellular aging[6][7]. The role of IRS methylation is to control genome stability. Loss of Alu methylation in aging cells may lead to genomic instability, one of the hallmarks of aging cells. Cells of individuals with CUG may require higher levels of genome stability. Our PCR evaluated overall methylation statuses of a hundred thousand copies of Alu, but only a few thousand copies of LINE-1. Although there are hundreds of thousands of copies of Alu and LINE-1, most of LINE-1 are truncated and missing CpG dinucleotide containing 5’UTR. Therefore, Alu methylation represents genomic methylation more than LINE-1, consequently genomic stability.

This study is the first to show the correlation of IRS methylation in young adults with that of new born phenotypes. Therefore, it is highly likely that IRS methylation is quite stable. There are a number of studies that show differences in IRS methylation in WBC of many diseases [8–14]. Therefore, IRS methylation is a potential marker for disease risk prediction.

An additional study to evaluate Alu methylation levels at birth is useful to prove that Alu hypermethylation is discoverable at birth. Moreover, Alu methylation level may be useful in predict in growth rates of new born infants and better nutritional management. This is particularly important because many previous studies found CUG in early life is associated with metabolic syndrome [39][40] and future coronary artery disease [41][42]. Guenard and colleagues conduct a study to analyse the effect of maternal weight loss surgery (bariatric surgery) on methylation levels of genes involved in cardiometabolic pathway in before surgery and after surgery [43]. Based on 5,698 genes, the methylation level was differentiated between before surgery and after surgery sibling, indicating a preponderance of glucoregulatory, inflammatory and vascular disease genes. They also demonstrated previously that the prevalence of obesity, adiposity, hypertension, dyslipidemia in children born after bariatric surgery was markedly lower than in sibling born before maternal bariatric surgery[44]. They suggest that these improvements in cardiometabolic indicators may be attributable to an improvement intrauterine environment. Similarly our study found the mean level of Alu methylation was higher in CUG group than those non-CUG. Maternal in CUG had higher incidence of pregnancy induced hypertension and lower maternal diet of fat in first trimester than those in non-CUG group. Also CUG group was associated with higher waist circumference and BMI than those in non-CUG group in adult. These stressed the importance of the intrauterine environment such as nutritional factors and maternal stress in fetal programming [45]. Epigenetics is a potential mechanism of this association.

## Conclusions

This study showed the positive correlation between the history of CUG in the first year, and that higher Alu methylation indicates the role of Alu methylation in the human growth process. To our knowledge, this is the first study which demonstrated the association between a phenotype during the newborn period and IRS methylation during young adulthood. Knowing Alu methylation levels at birth may be useful in predicting the growth rate of newborns, and better nutritional management to prevent metabolic syndrome and coronary artery disease in adults.
